# Revisiting ecological fallacy: are single-case experimental study designs even more relevant in the era of precision medicine?

**DOI:** 10.1093/pcmedi/pbae025

**Published:** 2024-10-18

**Authors:** Theodoros Christophides, Maria Karekla

**Affiliations:** Department of Psychology, University of Cyprus Medical School, University of Cyprus, Nicosia 1678, Cyprus; Department of Cardiology, Nicosia General Hospital, State Health Services Organisation, Nicosia 1678, Cyprus; Department of Psychology, University of Cyprus, Nicosia 1678, Cyprus

Dear Editor,

“Give different ones [liquid medicines] to different patients, for the sweet ones do not benefit everyone, nor do the astringent ones, nor all the patients able to drink the same things”

–Hippocrates

(Diseases III, Vol VI)

It was >70 years ago that William S. Robinson cautioned against deducing conclusions on an individualistic level based on ‘ecological’ or population-level data [1]. Ecological fallacy, the potentially flawed inference from group or population research data to the individual, has historically been recognised as an important limitation encountered in a wide area of disciplines within the humanities, natural sciences, and medicine [2]. Given the increasing momentum of personalized therapeutic strategies, the ecological correlation problem remains highly relevant in the era of precision medicine.

Many literature references regarding limitations in cross-level inferences relate to observational-ecological research [2]. However, randomized controlled trials (RCTs), considered the gold standard for testing efficacy and effectiveness of intervention(s) between different groups, are not ‘exempt’ from bias, with ecological fallacy being one of the most well-known limitations with regards to their external validity [3]. It is often assumed that the findings of an RCT would be transferable to all populations, at different times, and in different treatment environments. However, to be able to improve internal validity by controlling for confounding variables and enhance statistical power, strict inclusion criteria are often set for recruitment, to obtain a homogeneous sample of diagnostically uniform participants [3]. This ‘artificial’ trial environment does not often match the real-world setting and the complexities of the patients seen in the everyday clinic. To put it simply, the ‘average patient’ may not always exist or in other words, a particular treatment may work for some patients who may drive the differences but may have no significant impact or may not work at all for others.

A good example to illustrate this is statin therapy, the cornerstone of lipid-lowering treatment, where despite the considerable body of evidence supporting the use of statins in multiple clinical settings, there is a very wide variability in response from real-world data, with suboptimal results [4]. A main reason for these observations relates to the unique genetic makeup of individuals, or gene polymorphisms, which have not been accounted for during trial design in earlier studies [4].

RCTs may thus not be the best trial design for all research needs and for studies that require diagnostic accuracy. Hence clinical applicability to the individual patient, as well as better reporting of harms, may be addressed more effectively by using a case-based approach that is however experimental in nature and can be used to infer causation, such as the single-case experimental design (SCED) study approach.

SCED refers to a group of research designs, where the central unit of analysis is the single case (individual, clinic, or community), with repeated measurements of variables as they vary in time, before, during, and after the intervention, to evaluate their effects (intrasubject variation) [5]. SCED thus approaches a research hypothesis from an idiographic perspective. Analysing further the logic behind SCED, data are not aggregated or averaged across subjects; they are analysed on a case-by-case basis, whilst also permitting comparison between participants, using multiple baseline designs. This allows for all the important details that individual data provide to be acknowledged and appreciated accordingly, so nothing of research value is lost during analysis. Furthermore, this approach avoids the risk of pattern searching in averages of homogenous groups and attempting a cross-level application of inferences, which inherently predisposes to ecological fallacy.

The above points can be illustrated again using the example of statins, and specifically their adverse effects. Blinded RCTs have reported almost no side-effects for patients on statins beyond placebo, yet in daily (‘unblinded’) clinical practice, there are frequent reports of patients experiencing significant side-effects [6]. Finegold *et al*., in a meta-analysis of blinded statin RCTs with >80 000 participants, demonstrated that patients more commonly had to stop taking placebo tablets than statins due to side-effects [7]. SCED studies have been utilized to find possible explanations for this paradox between the results from the RCTs and the experiences of individuals. Two such studies are the StatinWISE and SAMSON trials from the UK [8, 9]. Both trials found no significant difference between statins and placebo and were able to provide specific explanations for this observation. These included, apart from the pharmacologic effect, chronic background symptoms, intercurrent conditions which could potentially induce symptoms, and nocebo effects. Furthermore, in the SAMSON trial, approximately half of the patients (who previously experienced intolerable adverse reactions to statins), after appreciating their individual side-effect profile, were able to restart statins and thus re-establish a significant prognostic benefit for their cardiovascular health [6].

SCED studies, however, are not themselves without limitations. In a simplistic overview, these include (i) the continuous need for improvement of methodological guidelines for more robust investigational rigour, (ii) researcher and participant subjectivity (including the issue of autocorrelation when systematically sampling the same individual over time), and (iii) the generalizability of the results [10].

Nonetheless, it is tempting to consider an interesting notion, that of the integration of SCED studies into RCTs, whereby participants will be matched to specific treatment groups, rather than exposing all the members of a group to the same treatment. As reviewed in 2022 by Epstein and Dallery, this amalgamated study design can offer several advantages; firstly, it can allow for an initial ‘proof-of-concept’ of novel treatments compared to the existing standards. Furthermore, it can theoretically improve adherence and reduce drop-out rates for participants, hence increasing the study power, an important advantage in the case of small sample studies. Finally, it can potentially reduce variability in outcomes for participants who have been ‘matched’ to specific treatments using an initial SCED study [5].

Thus, from the above, the SCED can provide a novel approach to personalized treatments, with detailed individual data sets constructed for each patient, ultimately promoting a working alliance between the patient and the physician to come to a personalized solution, and thus increase treatment efficiency and effectiveness. This is the essence of precision medicine.
